# The implementation of expectancy-based strategic processes is delayed in normal aging

**DOI:** 10.1371/journal.pone.0214322

**Published:** 2019-03-25

**Authors:** Carmen Noguera, Sergio Fernández, Dolores Álvarez, Encarna Carmona, Paloma Marí-Beffa, Juan J. Ortells

**Affiliations:** 1 Department of Psychology, University of Almería, Almería, Spain; 2 School of Psychology, University of Wales Bangor, Bangor, Gwynedd, United Kingdom; Baycrest Health Sciences, CANADA

## Abstract

The present research examined if the time needed to implement expectancy-based strategic processes is different in younger and healthy older adults. In four experiments participants from both age groups performed different strategic priming tasks. These included a greater proportion of incongruent (or unrelated; 80%) than of congruent (or related; 20%) trials. With this procedure performance is worse for congruent (less frequent) than for incongruent (more frequent) trials, thus demonstrating that the relative frequency information can be used to predict the upcoming target. To explore the time course of these expectancy-based effects, the prime-target SOA was manipulated across experiments through a range of intervals: 400, 1000 and 2000 ms. Participants also performed a change localization and an antisaccade task to assess their working memory and attention control capacities. The results showed that increases in age were associated with (a) a slower processing-speed, (b) a decline in WM capacity, and (c) a decreased capacity for attentional control. The latter was evidenced by a disproportionate deterioration of performance in the antisaccade trials compared to the prosaccade ones in the older group. Results from the priming tasks showed a delay in the implementation of expectancies in older adults. Whereas younger participants showed strategic effects already at 1000 ms, older participants consistently failed to show expectancy-based priming during the same interval. Importantly, these effects appeared later at 2000 ms, being similar in magnitude to those by the younger participants and unaffected by task practice. The present findings demonstrate that the ability to implement expectancy-based strategies is slowed down in normal aging.

## Introduction

It well established that many aspects of cognition decline with normal aging, including attention, working memory (WM), and episodic memory [1, 2]. In an attempt to provide a potentially unifying underlying mechanism that could explain the diversity of age-related cognitive deficits, several alternative hypotheses have been proposed.

An influential account is the processing speed hypothesis of cognitive aging [3], which attributes age-related cognitive decline to a general slowing of information processing. This slowing in cognitive processing speed has been found for example in several perceptual speed tasks involving visual search, elementary comparison, and substitution operations [4, 5].

Another leading alternative, though not incompatible hypothesis suggests that cognitive deficits associated with aging would mainly be the result of an inability to inhibit or control for interference from task-irrelevant (external or internal) information [6–8]. Evidence supporting the inhibitory account of cognitive aging comes from a variety of experimental tasks thought to draw on inhibitory (top-down) control. Thus, older adults usually display less efficient inhibition of dominant but inappropriate reactions, as it is for example the case in the antisaccade task [9]. Performance by older adults is also poorer than for younger ones on WM tasks that require actively holding the relevant information in an easily accessible form, especially in the face of distraction or interference [10, 11].

Older adults can also show impoverished inhibitory memory control relative to younger subjects in different episodic memory tasks (e.g., intentional or directed forgetting [12, 13]), as well as in selective attention tasks that require active rejection of distracting information. This can explain why interference effects from irrelevant distractors in conflict tasks (e.g., Stroop; Eriksen-type flanker) are usually increased in old age [14–17]. In a similar vein, older adults, like younger subjects with lower WM capacity (WMC), can have greater difficulty to prevent or suppress the processing of to-be-ignored distractors in negative priming tasks [16, 18, 19].

On the other hand, several other lines of evidence suggest that the development of facilitatory strategies of the type required to consciously expect forthcoming targets would also be affected by a reduced availability in WM resources, as it could be the case in older people [20– 23], or in younger adults with a lower WM capacity. In this later case, some recent priming studies have shown that, when the manipulation promotes the generation of expectancy-based strategies (e.g., with long prime-target interval; increased proportion of related pairs), these controlled semantic priming effects can be significantly diminished (or even eliminated) for participants with low attention control, low WMC [24, 25], or under high WM load [26–28]. Several neuroimaging studies report in fact evidence that Prefrontal cortex (PFC), an area (along with the anterior cingulate cortex) known to reflect attention control [29], is highly active when semantic information has to be generated and maintained over a delay in working memory [30, 31], or during semantic priming tasks performed under strategic conditions (i.e., a high relatedness proportion) [32, 33].

Note however that most of previous work investigating a possible dependence of strategic priming on WM resources, has used a conventional facilitation paradigm in which both the controlled and automatic processes (e.g. expectancy generation vs spreading of activation) produce the same behavioral pattern, that is, improved performance or facilitatory priming. Therefore, results from these studies showing changes in facilitation cannot be fully to be attributed to one process or another. Recently, we have developed an alternative priming task with the idea of producing qualitatively different behavioral effects depending on whether the processing of information is strategic or not [27–28]. To do so we used a Stroop-priming task in which a prime word (GREEN or RED) was followed by a coloured target (red vs. green) that participants had to identify. The prime word and target color were congruent or incongruent on 20% and 80% of the trials respectively, with instructions highlighting this proportion manipulation. The greater proportion of incongruent trials should bias participants to respond to the color opposite to that referred by the prime word. This anticipatory strategy should counteract the impact of automatic word reading, resulting in a strategic reversal of the Stroop effect with faster responses on incongruent than on congruent trials.

Importantly for the present goals, in one of the studies [27], the Stroop-priming task was interleaved with a concurrent verbal WM task demanding either a low load (memorizing a sequence of a same digit repeated five times), or a high load (retaining sequences of five different random digits). Ortells et al. found a substantial crossover interaction between prime-target congruency and working memory load. When participants performed the Stroop task under low WM load, a reliable reversed Stroop was observed demonstrating that they were able to strategically use the predictive information provided by the prime word to anticipate the color target. In clear contrast, under a high WM load the opposite standard Stroop interference effect was rather found (i.e., slower responses on incongruent than on congruent trials). These findings thus provide further evidence that the availability of working memory is crucial for implementing expectancy-based strategic actions.

Particularly relevant to the present research is a study by Froufe et al. [20], in which a group of healthy younger participants, and two groups of older adults, one with and one without Alzheimer’s dementia (AD), performed a Stroop-priming task very similar to that recently used by Ortells et al. [27, 28]. The proportion of incongruent trial was much higher (84%) than for the congruent ones (16%); but this time participants did not have to perform a concurrent WM task. Similar to what was found by Ortells et al. [27, 28] under low WM load, the younger group in Froufe et al. study [20] showed a reliable reversed Stroop effect. This ability of young adults to generate expectancy-based strategies has also been reported in other previous studies using similar strategic priming tasks [34–38]. On the contrary, the group of AD patients showed the opposite Stroop interference effect (i.e., slower responses on incongruent than on congruent trials), which suggests an inability in these patients to generate these expectations. Interestingly, the group of healthy older adults did not show Stroop interference effect, but they showed neither a reliable reversed Stroop, thus suggesting that their expectations were not effective enough to counteract the automatic interference.

Note that the prime-target stimulus onset asynchrony (SOA) interval used by Froufe et al. [20] in their study (1125 ms) was long enough to develop expectancy-based controlled strategies. However, as previously mentioned, if aging brings an overall decline in processing speed, then this interval may not be always enough for older adults. In support of this, several studies have demonstrated that the ability to suppress the processing of irrelevant information (at least in WM tasks) does not vanish with normal aging, but is delayed. This points to a potential interaction between deficits in inhibitory control and processing speed in older people [10, 39, 40].

Based on these considerations, one could argue that the ability to generate expectations could also be delayed in older adults. This could explain the absence of a reliable strategic (reversed Stroop) effect observed by Froufe et al. [20] in the healthy older group, despite using a seemingly long prime-target SOA. This lack of expectancy-based effects in healthy older adults has also been reported with other semantic priming tasks using relatively long SOAs (i.e., 950 ms) [21]. Unfortunately, the prime-target SOA was not manipulated in these studies. Alternatively, it is also possible that this lack of strategic indexes a reduction in cognitive control capacities. In any case, both Froufe et al. [20] and Langley et al. [21] did point to a potential reduction in WM and/or attentional control in the older group; but did not directly measured any of them, becoming this one of the main goals of our study.

### Current study

The main aim of the present study was to explore if the time needed to implement expectancy-based strategic processes with full efficiency is different in younger and healthy older adults. To this end, participants from both age groups performed different strategic priming tasks, across different experiments, with the purpose to dissociate between priming effects resulting from a controlled (strategic) vs. non-controlled (automatic) processing of critical stimuli [20]. This time, however, we used different prime-target SOAs to examine the time course of the strategic processes. In addition, in all our experiments, participants from the two age groups also performed two further tasks to assess their WM and attention control capacities.

## Experiments 1 and 2

In these experiments we used a similar version of the Stroop-Priming task previously employed by Froufe et al. (2009) [20]. This time however, we manipulated different prime-target SOAs in both Experiment 1 (400 vs. 1000 ms-SOA) and Experiment 2 (1000 vs. 2000 ms-SOA) to investigate directly the time course of expectancy-based strategic processes. We included a short prime-target SOA of 400 ms to examine if younger participants show a reversed Stroop (strategic) effect even at that relatively short SOA interval. Using a similar Stroop-Priming task, previous research has reported strategic effects by young adults at relatively short SOAs of 300–400 ms [36].

On the other extreme, we decided to include a long SOA of 2000 ms (instead of 1125 ms as in Froufe et al. [20]) to give older adults enough time to fully implement controlled strategies if that was the problem. However, the control deficits affecting older adults may not be solely linked to poor processing speed. Some authors have suggested that they may also stem from difficulties maintaining relevant information in WM. For example, expectancy effects in younger adults usually increase with the SOA, but this is not always the case for the older ones. In some priming studies where the prime stimulus does not remain on screen during the interstimulus interval, older adults display a decline in expectancy effects when the SOA increased [41]. A possible interpretation of all these findings is that that older and younger individuals may differ in both the speed needed to implement strategies and in the capacity to maintain relevant information in WM. Including a SOA of 2000 ms will allow testing for both possibilities.

All participants in our experiments also completed (a) the Change Localization task [28, 42], and (b) a version of the Antisaccade task [25, 43, 44]. In the Change Localization task, a sample array containing four colored circles is followed by a test array. This one is identical to the sample array except for one of the four circles presented in a different color. The task is to report the location of the change. The simplicity of this task is in sharp contrast with other more conventional WM span tasks [45] providing additional advantages. For example, it is very short -between 8 and 10 minutes-, there is no time pressure, no task switching, and there is no need for background knowledge (e.g., vocabulary or math facts) to perform it. For all these reasons, this task is especially well fit to assess WM in older people and in some clinical populations [42]. The fact that chance is 25% rather than 50% also minimizes guessing effects and increases measurement reliability [42]. Importantly, despite its simplicity, its validity is comparable to other more complex measures of WM capacity (e.g., Operation Span task), correlating strongly with general measures of higher cognitive abilities (including fluid intelligence) and attention control in both healthy adults and clinical populations (e.g., people with schizophrenia) [42, 46–48].

In the Antisaccade task, participants have to identify a letter target briefly presented at the left or right of fixation. The target is preceded by an abrupt-onset cue appearing either on the same (prosaccade block), or on the opposite side of fixation (antisaccade block). In the prosaccade trials, participants benefit from the appearance of the cue, which automatically oriented attention towards the target location. Unlike the prosaccade trials, in the antisaccade trials, participants have look away from the flashed cue to be able to identify the target on the opposite visual field before it disappeared. This ability to reorient from the cue to the target location seems to be dependent on WMC. Previous work with the antisaccade task has reported differences in groups supposedly varying in WMC, such as schizophrenics or patients with lesions in the prefrontal cortex when compared to healthy controls [49]; older compared to younger adults [50], or even between younger adults varying in WMC [19, 25, 43, 44].

The inclusion of a prosaccade condition in our task, which is more dependent on automatic orienting of attention (thus requiring less executive control and WMC), would allow to assess the differential performance between the prosaccade and antisaccade conditions, as an additional index of attentional control. If older adults have mainly a general decline in speed, then responses should be slower in both prosaccade and antisaccade trials. Conversely, if they have also a decreased attentional control capacity then their performance could be much worse on the antisaccade than on prosaccade trials (i.e., a reliable interaction between Age Group and Saccade Condition -antisaccade vs. prosaccade).

Lastly, older adults in the two Experiments were also pre-screened to rule out cognitive impairment. More detailed information about these tests can be found in the next section.

### Materials and method

#### Participants

Fifty-two (26 young and 26 older adults) and fifty-two (26 young and 26 older adults) native Spanish speakers with normal or corrected-to-normal vision participated in Experiments 1 and 2, respectively. These sample sizes were similar to that used by previous studies addressing strategic priming (e.g., Froufe et al., 2009 [20]; n = 27; Ortells et al. [27]; n = 26). The younger groups were undergraduate students from the University of Almería [Experiment 1: mean age = 21.2; SD = 3.7; range = 18–32; 15 females; Experiment 2: mean age = 22.3; SD = 2.7; range = 18–29; 14 females] who received course credits for their participation. Participants in the older groups were healthy volunteers recruited through the ‘University for the Older People’ Learning Program developed by the University of Almería [Experiment 1: mean age = 70.5; SD = 4.2; range = 65–78; 14 females; Experiment 2: mean age = 70; SD = 2.7; range = 65–75; 14 females]. All the experiments of the present research were conducted in compliance with the Helsinki Declaration, and with the ethical protocols and recommendations of the “Code of Good Practices in Research”, “Commission on Bioethics in Research from the University of Almería”. Participants also signed informed consents before their inclusion, with the protocol being approved by the “Bioethics Committee in Human Research” from the University of Almería.

The older participants were prescreened to ensure that they were healthy with no history of neurological, psychiatric or vascular disease, showed no symptoms of depression, nor were taking any psychoactive drugs or medication for high blood pressure. These tests were taken at the beginning of each experimental session and consisted of: (a) the 35-point Lobo’s Mini-Examen Cognoscitivo (MEC; [51]), a validated Spanish version in the elderly population of Mini-Mental State Examination [52], used to rule out cognitive impairment or dementia (norm score > 24); (b) the Spanish version [53] of the Yesavage abbreviated questionnaire (GDS; [54]), employed to test for depression (norm score < 5); (c) the Lawton and Brody Instrumental Activities Of Daily Living Scale (IADL; [55]), validated to Spanish [56], to measure the degree of autonomy of the person in different daily activities crucial for independent living (norm score > 7). None of the older adults were excluded from the experiments, since all scored within two standard deviations of the norm on each of the above tests (see Table 1).

**Table 1:** Screening scores for the older participants in Experiments 1 to 4.

	Experiment 1 Mean (SD)	Experiment 2 Mean (SD)	Experiment 3 Mean (SD)	Experiment 4 Mean (SD)
**MEC**	32.62 (1.55)	32.96 (1.61)	33.54 (1.07)	33.35 (0.94)
**GDS**	0.69 (1.01)	0.58 (0.99)	0.65 (0.85)	0.69 (0.84)
**IADL**	7.88 (0.33)	7.92 (0.39)	7.92 (0.39)	7.81 (0.49)

#### Apparatus and stimuli

The experiments were run on a PC using E-Prime software v2.0 (Psychology Software Tools, Pittsburgh, PA). Stimuli were displayed on a 17-inch CRT monitor at a viewing distance of approximately 60cm. Responses were collected using a standard keyboard.

The Change Localization task trials consisted of four colored circles subtending about 0.96° horizontally and 0.96° vertically. The four colors were randomly selected from a set of nine different colors with the following red, green, and blue (RGB) values: orange (255, 113, 0), yellow (255, 255, 0), magenta (255, 0, 255), red (255, 0, 0), white (255, 255, 255), blue (0, 0, 255), black (0, 0, 0), cyan (0, 255, 255), and green (0, 255, 0). The circles were displayed forming a circumference. The distance between fixation and the nearest and farthest stimuli subtended 3.36° and 6.24°, respectively. All stimuli were displayed against a grey background (60, 60, 50).

In the Antisaccade task, the target stimulus consisted of the letters “O” or “Q” (in Courier new font size 22) subtending about 0.43° horizontally and 0.86° vertically. They were displayed in white against a black background at a distance of 3° to the left or right of fixation (+). The pattern mask following the target offset consisted of the characters “$$$$” (in Arial font size 22) over an area of 0.86° vertically and.43° horizontally.

In the Stroop-Priming task, the prime stimuli were two color words (RED or GREEN) in Courier new font size 22 written in white, with each letter occupying an area of about 0.35° wide and 0.52° high. The target was a rectangle presented in either red (255, 0, 0) or green (0, 255, 0) at fixation, subtending 7.39° horizontally and 2.6° vertically. All stimuli were presented against a black background.

#### Procedure

In both experiments participants from the two age groups did the Change Localization and the Antisaccade tasks (the order of both tasks was counterbalanced across participants) before performing the Stroop-Priming task. No effects involving task order approached significance in neither Experiment.

**Change-Localization task**. Each trial of this task began with a 1000 ms central fixation cross (+), which remained on the screen throughout the trial. The fixation was followed by a sample array presented for 150 ms consisting of four circles each one in a different color. After a 900 ms blank screen, a test array appeared, identical to the sample array except for one of the four items which was in a changed color. The task was to indicate the location of the change using the computer mouse. Each participant completed 12 practice trials followed by 64 experimental trials divided in two consecutive blocks (32 trials each) with time for a break between blocks.

**Antisaccade task**. Trials began with a white fixation cross (+) presented on a black background for a random duration between 500 and 1500 ms. A white asterisk (*) then appeared randomly 3.8° to either the left or right of fixation for 200 ms, followed by a 100 ms blank interval. A letter target (O vs. Q) then appeared on either the same side of the asterisk (prosaccade block) or the opposite one (antisaccade block). The target was displayed for 100 ms and was immediately followed by a pattern mask (##) of 5000 ms duration until response. Participants had to press either the ‘1’ or the ‘2’ keys on the computer keyboard to indicate the identity of the target, with key-target allocations being counterbalanced between participants. Participants completed two consecutive blocks of 64 trials/block (16 practice followed by 48 experimental trials), one for the antisaccade and other for the prosaccade block, with the order of blocks counterbalanced across participants. The block order did not reach significance in neither Experiment.

**Stroop-Priming task.** Each experimental trial began with a central fixation cross (+) presented for 500 ms, followed by the prime word “VERDE” (GREEN) or “ROJO” (RED) presented in white letters for 200 ms. The prime display offset was followed by blank screen for either 200, 800, or 1800 ms (depending on the prime-target SOA manipulation). The target was a colored rectangle in either green or red appearing at fixation. This remained on the screen 2000 ms or until response, whichever happened first (S1 Fig). The participants responded to the color (red or green) of the rectangle by pressing the keys ‘1’ or ‘2’ on the computer keyboard. Both keys were labeled RED and GREEN (with red and green stickers, respectively), with the location of the label counterbalanced between participants. Incorrect responses were followed by a 500-ms feedback emoticon (sad face). A 500 ms blank inter-trial interval was presented before the beginning of the next trial. The prime-target pairings were congruent (i.e., GREEN-green) on 20% of the trials and incongruent (i.e., GREEN-red) on the remaining 80%. Before the beginning of the experiment, participants from both age groups were explicitly informed about the differential proportion of congruent and incongruent prime-target pairings, and were actively encouraged to capitalize on the predictive information provided by the prime word to optimize their performance. Thus, they were told that given a particular prime word (e., RED), they should expect that the forthcoming color target would be the color not named by the prime (e.g., green), as incongruent prime-target pairs were much more frequent than congruent pairs.

There were 40 practice trials (20 for each prime-target SOA condition) followed by 120 experimental ones divided in two blocks: 60 trials for each prime-target SOA condition (Experiment 1: 400 and 1000 ms; Experiment 2: 1000 and 2000 ms), with the order of the blocks counterbalanced across participants. Within each SOA block, there were 48 incongruent trials (80%) and 12 congruent trials (20%), and the target (colored rectangle) was displayed in red or green the same number of trials. The participants initiated each prime-target block by pressing the space bar on the computer keyboard. Once a SOA block was initiated it ran to completion, so that the participants could rest only between blocks. Because the order of SOA blocks did not interact with any other variable in our Experiments, the data are collapsed across SOA order in the Results and Discussion section.

### Results and discussion

#### WM capacity and attention control tasks

To quantify WM storage Capacity (WMC) using the Change Localization task, a variant of the Pashler/Cowan K equation was used, where *K* represented how many items have been stored in WM [46]. Given that each trial contains a change, there is no potential for false alarms. As a result, *K* was calculated by multiplying each participant proportion of correct responses by four (the number of items in the memory array).

Table 2 presents descriptive statistics for young and older participants in both experiments for the Change Localization and the Antisaccade tasks. Independent-samples t-tests were used to examine whether there were significant differences in performance between younger and older participants for each task. As it can be seen in this Table, younger adults showed a significantly higher WM storage capacity than older adults in both Experiments 1 and 2. The younger group also responded reliably faster and more accurate than the older group in both the antisaccade and prosaccade trials.

**Table 2:** Comparisons between younger and older adults in Experiments 1 and 2.

	Younger mean (SD)	Older mean (SD)	Group differences	Effect Size *d*
**Experiment 1**				
Age	21.2 (3.7)	70.5 (4.2)	t (50) = 44.4	12.5
WMC	3.25 (.35)	2.05 (.44)	t (50) = 10.8	3.02
Antisaccade				
RT (ms) AC (%)	727 (259.6) .78 (.11)	1352 (537.2) .59 (.10)	t (36)^a^ = 5.34 t (50) = 6.7	1.48 1.81
Prosaccade				
RT (ms) AC (%)	579 (144.1) .88 (.08)	1044 (323.8) .74 (.10)	t (35)^a^ = 6.7 t (50) = 5.6	1.85 1.55
**Experiment 2**				
Age	22.3 (2.7)	69.6 (2.7)	t (50) = 63.4	17.5
WMC	3.17 (.42)	2.27 (.57)	t (50) = 6.4	1.80
Antisaccade				
RT (ms) AC (%)	610 (148.5) .84 (.10)	1245 (552.8) .61 (.12)	t (29)^a^ = 5.7 t (50) = 7.7	1.57 2.10
Prosaccade				
RT (ms) AC (%)	528 (132.3) .92 (.06)	955 (290.5) .75 (.13)	t (35)^a^ = 6.8 t (36)^a^ = 5.8	1.90 1.70

All *p* values < .0001

^a^ Correction of *df*s for unequal variances

Participants’ performance in the Change Localization and Antisaccade tasks was also assessed by correlation analyses, producing similar results for both experiments. Namely, WM Capacity and participants’ age were highly correlated [Experiment 1: *r* = -.86, *p* < .001; Experiment 2: *r* = -.70, *p* < .001], showing that increases in age are associated with a decline in WM Capacity. Participants’ WMC also correlated with their performance in both the antisaccade [Experiment 1 = RTs: *r* = -.69; AC %: *r* = .65; Experiment 2 = RTs: *r* = -.57; AC %: *r* = .77] and prosaccade trials [Experiment 1 = RTs: *r* = -.72; AC %: *r* = .55; Experiment 2 = RTs: *r* = -.49; AC %: *r* = .72], as well as with the difference between antisaccade and prosaccade conditions [Experiment 1: RTs: *r* = -.45, *p* = .001; AC %: *r* = —.31, *p* = .024; Experiment 2: RTs: *r* = -.39, *p* = .004; AC %: *r* = —.32, *p* = .02). This latter finding suggests that the reduced WMC showed by older adults in the two experiments (as compared with younger participants) seems to be associated with two main causes. First, a slower processing-speed, demonstrated by increased reaction times in all conditions, including the prosaccade. And second, a decreased capacity for attentional control, as revealed by a disproportionate deterioration of performance in the antisaccade trials -compared to the prosaccade ones- in the older group.

These impressions were further confirmed using a mixed analysis of variance (ANOVA) in which Age (younger vs. older) was treated as a between-participants factor, and Saccade Type (antisaccade vs. prosaccade) as the within-participants variable. As expected, both Age [Experiment 1 [RTs: *F* (1, 50) = 36.5, *p <* 0.001, *η*^*2*^ = 0.42; Accuracy: *F* (1, 50) = 48.5, *p <* 0.001, *η*^*2*^ = 0.49; Experiment 2 [RTs: *F* (1, 50) = 47.1, *p <* 0.001, *η*^*2*^ = 0.48; Accuracy: *F* (1, 50) = 53.2, *p <* 0.001, *η*^*2*^ = 0.52], and Saccade Type were significant [Experiment 1 [RTs: *F* (1, 50) = 45.5, *p <* 0.001, *η*^*2*^ = 0.48; Accuracy: *F* (1, 50) = 101.8, *p <* 0.001, *η*^*2*^ = 0.67; Experiment 2 [RTs: *F* (1, 50) = 15.4, *p <* 0.001, *η*^*2*^ = 0.24; Accuracy: *F* (1, 50) = 107.6, *p <* 0.001, *η*^*2*^ = 0.68], and more interestingly, so it was the interaction between these two variables in both Experiment 1 [RTs: *F* (1, 50) = 5.6, *p =* 0.022, *η*^*2*^ = 0.10; Accuracy: *F* (1, 50) = 3.4, *p =* 0.07, *η*^*2*^ = 0.06] and Experiment 2 [RTs: *F* (1, 50) = 4.8, *p =* 0.03, *η*^*2*^ = 0.09; Accuracy: *F* (1, 50) = 9.02, *p =* 0.04, *η*^*2*^ = 0.15].

#### Stroop-Priming task

Trials containing an incorrect response (Experiment 1 = 1.4%; Experiment 2 = 1.45%), or those with reaction times (RTs) faster than 200 ms or falling more than 2.5 standard deviations from the overall mean RT (Experiment 1 = 1.1%; Experiment 2 = 1.9%) were removed from analyses.

Mean correct RTs and error rates were computed for each participant in the two age groups as a function of Congruency and SOA. Results were analyzed with a 2 (Congruency: congruent, incongruent) x 2 (SOA: -Experiment 1: 400, 1000-, -Experiment 2: 1000, 2000) x 2 (Age Group: younger, older) mixed ANOVA for each Experiment, with Congruency and SOA manipulated within participant and Age between groups (see Table 3; values in parentheses are Standard Deviations).

**Table 3:** Responses in the Stroop-Priming task for Experiments 1 and 2.

		Prime-target Congruency			
		Younger		Older	
		Congruent	Incongruent	Congruent	Incongruent
**Exp 1**	400-ms	527 (101.1) 1.1 (2.7)	562 (97.3) 1.4 (1.7)	784 (181.5) 1.7 (2.9)	826 (197.4) 2.4 (3.1)
	1000-ms	579 (172.9) 1.1 (2.3)	543 (133.8) 1.1 (2.3)	778 (176.2) 1.2 (2.9)	780 (181.1) 1.2 (2.9)
**Exp 2**	1000-ms	553 (151.1) 1.5 (2.9)	514 (122.7) 1.4 (1.9)	720 (164.3) 1.5 (2.9)	726 (161.8) 1.6 (2.3)
	2000-ms	554 (139.7) 1.4 (2.1)	513 (119.1) 1.2 (2.1)	751 (140.7) 1.5 (2.9)	711 (128.6) 1.4 (1.7)

Error rates showed no significant effect in either Experiment (all *p*s > .24). The RTs revealed a main effect of Age Group in both Experiment 1 [*F* (1, 50) = 33.3, *p <* 0.001, *η*^*2*^ = 0.40], and Experiment 2 [*F* (1, 50) = 26.3, *p <* 0.001, *η*^*2*^ = 0.34], where older participants were slower (Experiment 1 = 792 ms; Experiment 2 = 727 ms) than the younger ones (Experiment 1 = 553 ms; Experiment 2 = 533). The interaction between SOA and Congruency was also significant in the two experiments [Experiment 1 = *F* (1, 50) = 58.2, *p <* 0.001, *η*^*2*^ = 0.54; Experiment 2 = *F* (1, 50) = 5.01, *p =* 0.03, *η*^*2*^ = 0.09], and even more relevant was the three-way significant interaction between SOA, prime-target Congruency, and Age Group [Experiment 1 = *F* (1, 50) = 4.35, *p =* 0.04, *η*^*2*^ = 0.08; Experiment 2 = *F* (1, 50) = 4.20, *p =* 0.046, *η*^*2*^ = 0.08]. Further analyses revealed a very different Stroop-Priming pattern as a function of prime-target SOA for the two age groups in each experiment (S2 Fig).

In Experiment 1 (400 and 1000 SOA), the younger group showed the opposite Stroop-Priming pattern for each SOA, (Congruency x SOA interaction, [*F* (1, 25) = 34.01, *p <* 0.001, *η*^*2*^ = 0.58]). Namely, we observed a standard Stroop interference at 400-ms SOA (-35 ms; *F* (1, 25) = 5.9, *p =* 0.02, *η*^*2*^ = 0.19), and a reversed (strategic) Stroop at the longer 1000-ms SOA (+36 ms; *F* (1, 25) = 9.46, *p =* 0.005, *η*^*2*^ = 0.28). The Congruency x SOA interaction was also evident in the older group (*F* (1, 25) = 25.1, *p <* 0.001, *η*^*2*^ = 0.50), presenting standard Stroop interference at 400ms SOA (-42 ms; *F* (1, 25) = 23.5, *p <* 0.001, *η*^*2*^ = 0.49). However, unlike the younger group, neither standard nor reversed strategic effects were observed at the longer 1000ms SOA (-2 ms; *F* < 1). This finding closely replicates the absence of reversed Stroop reported by Froufe et al. [20] with healthy older people under very similar task conditions.

In Experiment 2 (1000 and 2000 SOA), the youngest exhibited strategic reversed Stroop in both 1000 ms (+39 ms; *F* (1, 25) = 9.4, *p =* 0.005, *η*^*2*^ = 0.27) and 2000 ms SOA (+41 ms; *F* (1, 25) = 22.1, *p =* 0.001, *η*^*2*^ = 0.47). In contrast, in the older group we found a reliable Congruency x SOA interaction (*F* (1, 25) = 14.3, *p =* 0.001, *η*^*2*^ = 0.36). At the SOA of 1000 ms there was no reliable effect of any kind (-6 ms; *F* < 1), replicating what found in Experiment 1. But at the longest 2000 ms SOA, they showed reliable strategic effects of a similar magnitude to those found in the younger group (+40 ms; *F* (1, 25) = 32.4, *p <* 0.001, *η*^*2*^ = 0.56).

The lack of a reliable strategic effect (reversed Stroop) at the SOA of 1000 ms in both Experiments 1 and 2 closely replicates the findings reported by Froufe et al. [20] with healthy older people using very similar task conditions (i.e., SOA = 1150 ms). These results do not necessarily imply that all the older participants in our experiments were unable to show strategic effect at the SOA of 1000-ms. It is possible that some older adults can implement predictive strategies better and/or faster than others. Indeed, in a series of further correlation analyses for the entire sample of participants we found that the amount of strategic Stroop-priming (i.e., congruent minus incongruent) at the 1000-ms SOA condition was negatively correlated with age in both Experiment 1 (*r* = -.36, *p* < .007) and Experiment 2 (*r* = -.37, *p* < .006). Even more interesting, the strategic priming at 1000-ms SOA also positively correlated with their WMC scores. This correlation reached statistical significance in Experiment 2 (*r* = .34, *p* < .014), but not in Experiment 1 (*r* = .19, *p >* .18). Still, these observations are consistent with other studies linking WM resources with the development of predictive controlled strategies [24, 26–28].

The consistent and reliable reversed Stroop showed by older participants at the longest 2000-ms SOA in Experiment 2, clearly contrast with previous reports finding a drop in expectancy-based priming for older adults, at least when the prime stimulus did not remain on the screen during the interstimulus interval [41]. The results of Experiment 2 thus suggest that older individuals do not necessarily find it more difficult to maintain the relevant information in their WM, at least under relatively simple tasks as the one used here.

## Experiment 3

In Experiments 1 and 2, we found that, compared to younger participants, older adults not only had lower WM capacity and Attention Control, but also needed more time to efficiently implement expectancy-based strategies. In the present study, we wanted to replicate the differential time-course of strategic processes associated with age using a semantic priming task. One of the advantages of this task is that we can include a greater stimulus set, also requiring higher conceptual level (semantic) of representation than the word color Stroop task. To this aim, we used pictorial stimuli previously used in our lab [37, 38], which have been shown to produce qualitatively different (opposite) semantic priming effects depending on whether the processing of the prime stimuli is strategic (conscious) or not (automatic).

Participants made a semantic judgment task (animal vs. inanimate object) about a picture target preceded by a picture prime. On 80% of the trials (unrelated condition) the prime and target pictures belong to different semantic categories (e.g., DOG—fork; SPOON—cat), whereas on the remaining 20% of the trials (related condition) they are made of highly associated members from the same category (e.g. DOG–cat; SPOON–fork). Participants were again strongly encouraged to use the predictive information provided by the prime picture to improve their categorization performance. Thus, given a prime picture (e.g. a dog), they should expect that the upcoming target would belong to the opposite semantic category (i.e., an object), as the unrelated trials were much more frequent (80%) than the related trials [57]. To investigate the time-course of congruency priming effects, the prime-target SOA was manipulated at two levels: 400 and 1000 ms, as in Experiment 1.

### Materials and method

#### Participants

Twenty-six younger and 26 older adults participated. All of them were native Spanish speakers and had self-reported normal or corrected-to-normal vision. The younger participants were undergraduate students from the University of Almería [Age = 20.6; SD = 2.5; Range = 18–30; 14 females) who participated in the study in exchange by course credits. Older participants (age = 70.6; SD = 4.7; range = 64–81; 15 females) were healthy volunteers recruited through the ‘University for the Older People’ Learning Program from the University of Almería, following the same protocol as described in the Experiments 1 and 2.

#### Stimuli and procedure

Before performing the Congruency-priming task, participants from the two age groups performed the WM (Change Localization) and Attention Control (Antisaccade) tasks used in Experiments 1 and 2, with the order of tasks being again counterbalanced across participants (as in Experiments 1 and 2, there was no significant effect involving task order).

The picture stimuli were line drawings of 8 animals and 8 inanimate objects taken from the gray scale shaded images set by Snodgrass and Vanderwart [58]. They were displayed on a white background, with their dimensions ranging from 1.92° to 3.36° (height), and from 1.92° to 5.76° (width). For each participant and block of trials, each picture appeared five times as a prime and five times as a target stimulus. Each prime was paired with five different target pictures: one was a strongly associated co-exemplar (and highly similar in terms of feature overlap; see below), and four times were pictures from the opposite category.

All the semantically related pictures were also rated by participants in a previous similarity evaluation study as being highly similar in terms of both functional and visuals features. In that study, 80 picture pairs (40 animals and 40 inanimate objects) taken from the Snodgrass and Vanderwart [58]’ stimulus set, were presented to a different group of 100 undergraduate students from the University of Almeria. Participants had to rate both the functional and visual similarity of each picture pair (“In terms of features in common, how functionally and visually similar are the stimuli that these pictures refer to?”) on 7-point scales (1 = not at all similar; 7 = highly similar). Only those pairs from each category with rating scores higher than 5 points on the two 7-points scales were used for the congruency priming experiment (Appendix in S1 Appendix).

Each trial in the Congruency-Priming task began with a central fixation (a black cross) presented on a white background for a random duration between 500 and 1500 ms. A prime picture then appeared in the center of the screen for 200 ms, followed by a blank screen for either 200 ms or 800 ms (depending on the SOA condition). This was followed by a central target that remained on the screen 2000 ms or until response (S3 Fig). Participants indicated the semantic category (animal vs. inanimate object) of the target by pressing the ‘1’ and the ‘2’ keys on the computer keyboard, with key-category allocations being counterbalanced between participants. A 500-ms feedback emoticon (sad face) was presented following an incorrect response. A 500 ms inter-trial interval elapsed before the start of the next congruency-priming trial. The prime and target pictures belonged to different semantic categories (unrelated condition) on 80% of the trials, and they were semantically related co-exemplars (related condition) on 20% of the trials. As in the previous experiments, participants were explicitly informed about the differential proportion of related and unrelated prime-target pairings trials and were encouraged to use this for their own benefit.

Each participant took part in a single session (lasting about 20 min) consisting of 40 practice trials (20 for each SOA condition) followed by 160 experimental trials consisting of two consecutive blocks of 80 trials, one block for each SOA condition (400 ms vs. 1000 ms). The order of the two blocks was counter-balanced across participants (there was no significant effect involving SOA order). From the 80 experimental trials of each block, 64 were unrelated (80%) and 16 (20%) were related, and within each of these two trial-sets, the target picture belonged to either “animals” or “inanimate objects” category on the same number of trials.

### Results and discussion

#### WM capacity and attention control tasks

Descriptive statistics and comparisons between younger and older adults on WM storage capacity (Change Localization K-score), response speed (RTs) and accuracy (%) in the Attention Control task (antisaccade and prosaccade blocks) are presented in Table 4.

**Table 4:** Comparisons between younger and older adults.

	Younger Mean (SD)	Older Mean (SD)	Group differences*	Effect Size d
Age	20.6 (2.5)	70.6 (4.7)	t (38)^a^ = 48.04	13.3
WMC	3.24 (.38)	2.05 (.47)	t (50) = 10.1	2.80
**Antisaccade**				
RT (ms) AC (%)	622 (140.1) .80 (.90)	1327 (553.2) .56 (.09)	t (29)^a^ = 6.4 t (50) = 9.5	1.75 2.70
**Prosaccade**				
RT (ms) AC (%)	499 (93.6) .91 (.08)	1023 (373.5) .72 (.10)	t (28)^a^ = 6.9 t (50) = 7.0	1.92 2.10

*All *p* values < .0001 (Student’s t-test)

SD, Standard Deviations

^a^ Correction of *df*s for unequal variances

The differences found between younger and older adults were very similar to those in the previous experiments. Compared to their younger counterparts, older participants showed lower WM Capacity and responded slower, and less accurate, on both the antisaccade and prosaccade trials. As in Experiments 1 and 2, WMC and participants’ age were highly correlated (*r* = —.83, *p* < .001), again indicative of an age-related decline in WM Capacity. WMC scores also correlated with performance in the antisaccade [RTs: *r* = —.68; AC %: *r* = .82] and prosaccade blocks [*r* = —.69; AC %: *r* = .74], and also with the differences between the antisaccade and prosaccade conditions (RTs: *r* = —.47, *p* < .001; AC %: *r* = —.33, *p* = .02). As in Experiments 1 and 2, the results were further analyzed with a mixed ANOVA with Age as a between-participants factor and Saccade Type (antisaccade vs. prosaccade trials) as repeated measures. As previously found, there were reliable differences due to Age [RTs: *F* (1, 50) = 45.3, *p <* 0.001, *η*^*2*^ = 0.47; Accuracy: *F* (1, 50) = 76.9, *p <* 0.001, *η*^*2*^ = 0.61], and Saccade Type [RTs: *F* (1, 50) = 59.5, *p <* 0.001, *η*^*2*^ = 0.54; Accuracy: *F* (1, 50) = 156.2, *p <* 0.001, *η*^*2*^ = 0.76]. But more interestingly, there was a reliable interaction between these two variables in both RTs (*F* (1, 50) = 10.75, *p =* 0.003, *η*^*2*^ = 0.18) and Accuracy (*F* (1, 50) = 6.5, *p =* 0.014, *η*^*2*^ = 0.12). These results reinforce the idea that a reduction in WMC in older adults would be associated not only with a general response slowing, but also with a decreased capacity for attentional control.

#### Congruency-Priming task

For the analysis of responses in the Congruency-Priming task, we excluded trials with target responses incorrect (2.45%), and those with RTs faster than 200 ms, or falling more than 2.5 standard deviations from the overall mean RT (2.38%). Mean correct RT and error rates were computed for each participant in the two age groups as a function of Prime-Target Relatedness (related, unrelated) and SOA (400 ms, 1000 ms). These were further submitted to two separate 2 x 2 x 2 ANOVAs (different for RTs and error rates), with Relatedness and SOA as within participant manipulation, and Age (younger, older) as a between group factor (see Table 5).

**Table 5:** Results of the Congruency-Priming task for both age groups.

	Prime-target Relatedness			
	Younger		Older	
	Related	Unrelated	Related	Unrelated
400-ms	535 (70.8) 2.0 (2.8)	549 (80.9) 2.85 (2.5)	745 (207.1) 2.5 (3.4)	778 (174.1) 3.1 (3.5)
1000-ms	568 (94.5) 2.4 (3.5)	533 (94.1) 2.2 (2.2)	777 (208.6) 2.5 (3.9)	772 (180.5) 2.1 (3.6)

No effect was significant in the analysis of the error rates (all *p*s > .17). Conversely, ANOVA of RT data revealed a main effect of Age (*F* (1, 50) = 31.04, *p <* 0.001, *η*^*2*^ = 0.38), such that older participants responded again slower to the targets (768 ms) than the younger adults (546 ms). Prime-Target Relatedness interacted with both SOA (*F* (1, 50) = 19.4, *p <* 0.001, *η*^*2*^ = 0.28), and Age (*F* (1, 50) = 5.13, *p =* 0.028, *η*^*2*^ = 0.09). Further analyses of this later interaction revealed a very different priming pattern as a function of Age (S4 Fig). The younger adults showed a reliable relatedness x SOA interaction, (*F* (1, 25) = 25.9, *p <* 0.001, *η*^*2*^ = 0.51), due to a facilitatory priming effect at the shortest 400-ms SOA (+14 ms; *F* (1, 25) = 5.3, *p =* 0.03, *η*^*2*^ = 0.17), and an opposite (strategic) priming effect at 1000-ms SOA (-35 ms; *F* (1, 25) = 15.8, *p =* 0.001, *η*^*2*^ = 0.39). In clear contrast, the older group presented a facilitatory priming at the shortest 400-ms SOA (+34 ms; *F* (1, 25) = 5.1, *p =* 0.03, *η*^*2*^ = 0.17), but a lack of reversed (strategic) priming at 1000-ms SOA (-5 ms; *F* < 1), with a reliable Relatedness x SOA interaction in this Age group (*F* (1, 25) = 4.96, *p =* 0.03, *η*^*2*^ = 0.17).

The pattern across SOAs showed by older participants with this priming task, is consistent with what was found in Experiment 1 using a similar prime-target SOA interval with a different type of task (Stroop). Whereas younger adults are able to efficiently develop attentional strategies at SOA intervals on the range of 1000 ms, or even less (more than half of younger adults showed strategic priming effects in the Congruency-Priming task even in the shorter 400-ms SOA interval), the older group do not seem to be able to do the same within that interval. According to our previous results with the Stroop task, they seem to need longer for an efficient implementation of expectancy-based strategic processes.

As argued before (see Discussion section of Experiments 1 and 2), we do not discard the possibility that the older adults with a greater WMC could implement predictive strategies more effectively than others. Yet, the correlation between participants’ WMC and strategic priming at 1000-ms SOA did not reach statistical significance (*r* = .21, *p >* .126), as had occurred in Experiment 1.

## Experiment 4

This experiment had two main goals. First, to see if the expectancy-based strategic effecs found with a Stroop priming task at the longest 2000-ms SOA in older participants can also be found with other tasks, such as the congruency priming task used in Experiment 3. In this task the prime picture was also presented for a limited period of time (200 ms), being absent for the remaining SOA interval, as occurred in the Stroop-Priming task used in Experiments 1 and 2.

In addition, it has been argued that strategy-dependent processes may require some amount of practice until they can be implemented [38, 59, 60]. A second goal of this experiment was to investigate whether the strategic processing of goal-relevant information could be differentially modulated by task practice in older vs. younger adults. Previous work examining age and practice on cognitive performance have produced somewhat mixed findings. Some studies report that older adults have difficulties automatizing newly learned skills [61, 62]. Others, however, have found very similar practice effects in older and younger adults in different cognitive control tasks, such as task switching [63] or Stroop interference [64]. Yet, given that older adults generally show a reduced WMC and a slower processing speed than younger adults, it is possible that they also need more practice to be able to build these controlled strategies.

To test for that, here we used the same priming procedure used in Experiment 3 with two main differences: (a) the SOA remained fixed at 2000 ms; and (b) both young and older participants performed four consecutive blocks of trials. If the development of predictive strategies based on stimulus redundancy requires different amount of practice for young and older participants, then we expect to obtain a three-way interaction between Practice, Priming, and Age Group.

### Method

#### Participants

Twenty-six younger and 26 older adults participated in Experiment 4. All of them were native Spanish speakers self-reporting normal or corrected-to-normal vision. The younger participants were undergraduate students from the University of Almería (age = 22.1; SD = 4.2; range = 19–35; 15 females) who participated in the study in exchange by course credits. Older participants (age = 68.5; SD = 4.4; range = 64–81; 15 females) were healthy volunteers recruited through the same system described earlier.

#### Stimuli and procedure

These were similar to those used in Experiment 3, except that in the priming task (a) the animate and inanimate stimuli presented as primes were never presented as targets or vice versa; (b) the 200-ms prime picture was always followed by a blank screen of 1800 ms, such that the prime-target SOA remained fixed at 2000 ms; and (c) the task practice was manipulated, with participants from both age groups performing 4 consecutive blocks of experimental trials (40 trials per block). As in Experiment 3, within each block, the prime and target pictures belonged to different semantic categories on 80% of the trials (32), and they were semantically related co-exemplars on the 20% of the trials (8). Instructions emphasized the generation of category-based expectations as in previous experiments.

### Results and discussion

#### WM capacity and attention control tasks

Descriptive statistics for performance by both age groups in Change Localization and Antisaccade tasks are presented in Table 6, as well as comparisons between younger and older adults on WM storage capacity (Change Localization K-score) and response speed (RTs) and accuracy (%) in the Attention Control task (antisaccade and prosaccade blocks).

**Table 6:** Comparisons between younger and older adults in Experiment 4.

	Younger Mean	Older Mean	Group differences*	Effect Size d
Age	22.1 (4.2)	68.5 (4.4)	t (50) = 38.9	11.0
WMC	2.9 (.38)	2.3 (.55)	t (44)^a^ = 4.5	1.30
**Antisaccade**				
RT (ms) AC (%)	582 (119.1) .81 (.09)	1085 (515.4) .58 (.10)	t (28)^a^ = 4.8 t (50) = 8.7	1.34 2.40
**Prosaccade**				
RT (ms) AC (%)	520 (118.5) .89 (.07)	809 (290.3) .72 (.12)	t (33)^a^ = 4.71 t (41)^a^ = 7.1	1.30 1.97

*All *p* values < .0001 (Student’s t-test)

Values in parentheses are Standard Deviations.

^a^ Correction of *df*s for unequal variances

As in our previous experiments, WMC scores correlated again with age (*r* = -.56, *p* < .001), and with participants’ performance in the antisaccade [RTs: *r* = —.46; AC %: *r* = .79] and prosaccade trials [*r* = —.48; AC %: *r* = .74], as well as with the differences in performance between the both conditions (RTs: *r* = —.33, *p* = .02; AC %: *r* = —.36, *p* = .009), suggesting again a reduced capacity for attention control in older people. This conclusion is strengthened by results of further ANOVAs with Age (younger vs. older) and Saccade Type (antisaccade vs. prosaccade trials) as between- and within-participants variables, respectively. The main effects of Age [RTs: *F* (1, 50) = 28.4, *p <* 0.001, *η*^*2*^ = 0.33; Accuracy: *F* (1, 50) = 71.6, *p <* 0.001, *η*^*2*^ = 0.59] and Saccade Type were reliable again [RTs: *F* (1, 50) = 29.7, *p <* 0.001, *η*^*2*^ = 0.37; Accuracy: *F* (1, 50) = 143.8, *p <* 0.001, *η*^*2*^ = 0.74], as well as the interaction between these two factors, in both RTs (*F* (1, 50) = 11.82, *p =* 0.001, *η*^*2*^ = 0.19) and Accuracy (*F* (1, 50) = 6.7, *p =* 0.012, *η*^*2*^ = 0.12).

#### Congruency-Priming task

Trials containing an incorrect response (2.17% of trials), or with RTs faster than 200 ms or falling more than 2.5 standard deviations from the overall mean RT (2.10% of trials) were removed from analyses. Mean correct RT and error rates were computed for each participant in the two age groups as a function of task Practice (Trial Blocks 1–4), and Prime-Target Relatedness (Related, Unrelated). Resulting values were submitted to two different 4 x 2 x 2 ANOVAs, with task Practice and Prime-Target Relatedness as within participant variables, and Age (younger, older) as a between participants factor.

The ANOVA on error rates showed no significant effects (all *p*s > .28). The RT ANOVA revealed a main effect of Age (*F* (1, 50) = 29.2, *p* > 0.001, *η*^*2*^ = 0.37), as older adults were slower (694 ms) than younger adults (536 ms). We also found an overall effect of reversed priming (*F* (1, 50) = 37.8, *p* > 0.001, *η*^*2*^ = 0.43), because of faster responses to incongruent (590 ms) than congruent pairs (635 ms). Yet, this variable did not interact either with Practice or Age. Both younger and older participants showed very similar, and reliable, reversed priming effects, reaching significance from the first practice block for both age groups. Mean correct reaction times (in milliseconds) and error percentages (in %) in the Congruency-Priming task are presented in Table 7 (see also S5 Fig) as a function of Prime-Target Relatedness (Related vs. Unrelated), and Task Practice (Trial Blocks 1, 2, 3, and 4) across age groups (young vs. older participants).

**Table 7:** Responses in the Congruency-Priming task for each block.

	Prime-Target Relatedness			
	Younger		Older	
	Related	Unrelated	Related	Unrelated
Block 1	564 (117.8) 2.6 (4.8)	519 (92.9) 2 (2.8)	726 (180.3) 2.8 (5.1)	686 (132.3) 2.1 (3.1)
Block 2	565 (128.2) 2.4 (4.5)	526 (86.1) 2.2 (2.3)	723 (151.8) 2.8 (6.3)	678 (125.2) 2 (3.5)
Block 3	552 (94.9) 2.2 (5.7)	516 (88.2) 2.2 (2.9)	711 (127.1) 2.2 (4.4)	660 (106.5) 1.9 (2.4)
Block 4	536 (95.9) 2 (5.7)	514 (87.3) 1.7 (1.7)	701 (139.8) 2.1 (4.1)	667 (106.7) 1.6 (2.3)

Values in parentheses are Standard Deviations.

These results are consistent with those found in Experiment 2 using a different task, demonstrating once again that older adults, when given enough time, are able to implement controlled strategies as efficiently as younger adults do. Together, the findings of Experiments 2 and 4 suggest that older adults are able to maintain the relevant task information in their WM.

## General discussion

Cognitive impairment associated with normal aging has an impact in multiple domains, including selective attention, working memory, and episodic memory. Most of the deterioration seems to be rather specific of tasks involving controlled (top-down) processing [11]. This seems to be the case in those attention and memory tasks in which some irrelevant information interferes with target processing (arriving from internal or external sources) thus requiring some level of executive control to counteract it. Thus, it has been found that the presence of distracting information negatively impact task performance in older adults much more than in younger individuals [13, 15, 16, 39]. Interestingly, some recent research has suggested that in older adults, the ability to inhibit or supress irrelevant information could also be delayed in time [10, 39, 40].

The main goal of the present research was to investigate if the ability to develop task based expectancies is also delayed in normal aging. To this end, we conducted a series of four experiments where younger and older adults performed different strategic priming tasks. A common factor in these tasks was that the way prime information is used (strategic or controlled, vs. non-strategic or automatic) allows the measure of priming effects in opposite directions. To explore the time course of these effects, the SOA was manipulated across experiments and thus achieve a range of intervals, which included 400, 1000 and 2000 ms.

To further understand if these age differences were associated to changes in working memory capacity and attention control, both younger and older participants in our experiments also performed a Change Localization task (to assess WMC), and a version of the Antisaccade task that included both antisaccade and prosaccade trial blocks (to assess attention control).

This research strategy has produced a wealth of results. Firstly, relative to younger individuals, older adults responded consistently slower (and less accurate) in all the tasks. This result is consistent with a large body of evidence showing a generalized decline in processing speed with normal aging [3, 5]. A second relevant finding was a reduction in WMC in older adults found in all our experiments. Their performance in the Change Localization task was always worse than with younger participants. These results replicate the pattern found in other similar visual WM tasks, such as the Change Detection task [10].

An additional important finding was that performance in the WMC task (*k* scores) negatively correlated not only with the age or the overall speed in the Antisaccade task, but also with their differential performance between the antisaccade and prosaccade blocks. Thus, the decreased WMC likely resulting of aging seems to be associated not only with an overall processing speed decline, but also with a reduced capacity for attention control in older adults. This idea is further supported by evidence in all our experiments of older adults presenting greater deterioration of responses (both latency and accuracy) in the more control demanding antisaccade than in the prosaccade condition (see Tables 2, 4 and 6).

These findings are consistent with previous research showing that individuals with a lower WM Capacity are also impaired on several attention tasks, such as the Stroop or the antisaccade task, which require participants to maintain the relevant information in WM while performing an ongoing task [25, 29, 43]. Our results are also compatible with the executive attention theory of working memory developed by Engle and colleagues [65]. In that theory, inter-individual differences in WMC would mainly reflect variations in a domain-general attention control ability, necessary to sustain the task goal and constrain the focus of attention to relevant target in the presence of distraction.

Particularly relevant were the results from our priming tasks showing a delay in the development of expectancies in older adults. Younger participants showed strategic effects already at 1000 ms SOA (with Stroop in Experiments 1 and 2, with congruency priming in Experiment 3). But this was not the case with older participants, as they consistently failed to show reversed strategic priming during the same interval and tasks. These results replicate those found by Froufe et al. (2009) [20] in a similar strategic Stroop-like task with a prime-target SOA of 1125 ms. Other semantic priming studies using a similar SOAs (e.g., 950 ms) have also reported problems in Alzheimer’s Dementia and healthy older adults when having to generate attentional expectations for semantically related information [21]. One could therefore conclude that controlled strategic processing is impaired with normal aging.

Braver and colleagues have in fact suggested that one of the fundamental mechanisms that leads to age-related cognitive changes is a deficit in the ability to process contextual information for a proactive mode of control. These authors have developed the dual-mechanisms control (DMC) model to explain cognitive deficits in older adults and other clinical populations (e.g., schizophrenia). The model assumes that goal directed behavior could be the result of two different modes of cognitive control: proactive and reactive [66–68]. Proactive control would reflect a preparatory and resource demanding type of control in which a predictive cue (or context) is used by individuals to prepare a specific response to a future target. This control mode requires active maintenance of the goal-relevant information in an accessible state (in Working Memory) to efficiently focus attention on that information while ignoring competing distractors. In contrast to proactive control, the reactive form of control does not require a continuous effort or monitoring, but instead involves using a target stimulus to automatically retrieve appropriate actions from long-term memory. By using different tasks and experimental procedures (e.g., the AX-Continuous Performance Test, AX-CPT) to assess the DMC theory, Braver et al. have reported evidence that relative to younger individuals, healthy older adults would show a reduced tendency to use proactive control, but an increased tendency to use reactive control [68, 69].

On the other hand, as noted in the introduction, previous work has found that in younger adults expectancy effects are usally increased with the SOA, but they seem to decrease in older adults ([41]; Experiments 1 and 3), when the prime appears briefly on the screen and is not available during the interstimulus interval. That drop in expectancy effects could be due to difficutlies in the older group to maintain the relevant information in WM.

Note that in our priming tasks the prime stimulus did not remain on the screen during the whole SOA interval and, instead, was always presented for only 200 ms followed by a blank screen. If older participants in our experiments find it difficult to maintain information about the prime in WM compared to younger ones, then they may still show reduced strategic effects when giving them extra time (2000ms) to complete their strategies.

But this was clearly not the case in either Experiment 2 or Experiment 4. When the prime-target SOA was lengthened to 2000 ms, older adults could strategically use the information provided by the prime stimulus to anticipate the target response. They could hold this information in WM despite the prime stimulus disappearing after a short exposure until the target onset. Thus, our older participants responded reliably faster to incongruent than to congruent targets on both the Stroop task (Experiment 2) and the Congruency-priming task (Experiment 4). These strategic priming effects were similar in magnitude to those showed by the younger participants and were also unaffected by task practice. These results are in clear contrast to those reported previously by others [41] and the simplicity of our tasks might be the reason. Future research might be needed to investigate: a) how changes in WM load impact time course of expectancy generation in older adults, and b) how the perceptual availability of prime information during this period of time may reduce this load.

The present findings replicate and extend the results from other previous studies in showing that not only the ability to inhibit or disengage from irrelevant information, but also the ability to efficiently implement controlled facilitatory strategies (i.e., expectancy generation) would be delayed in time, rather than abolished in normal aging. An interesting issue for future research would be to compare strategic priming effects at longer SOA intervals (e.g., 2000 ms) in healthy older adults and those with Alzheimer’s dementia (AD). This would allow us to determine whether the deficits observed in the patient population are also due to a delay in the implementation of strategies (as observed with older adults) or instead, they are unable to generate predictive expectations even at intervals of 2000 ms or beyond.

## Supporting information

S1 Fig Stroop-Priming task. Examples of incongruent (left) and congruent (right) trials used in Experiments 1 and 2.
(TIF)

S2 Fig Stroop-Priming effects in Experiments 1 and 2. (TIF)

S3 Fig Congruency-Priming task. Sequence of events of an incongruent (left) and congruent (right) trial in the Congruency-priming task used in Experiments 3 and 4.
(TIF)

S4 Fig Congruency Priming effects in Experiment 3. (TIF)

S5 Fig Congruency Priming effects across Trial Blocks in Experiment 4. (TIF)

S1 Appendix List of prime-target pairs used in Experiments 3 and 4. Mean (SD) functional and visual similarity rates in a rating similarity pilot study (1 = not at all similar; 7 = highly similar), for animal and inanimate-object pictures (and their English translations), presented as semantically related prime-target pairs.
(PDF)
