# Supplementary figures and images for: The implementation of expectancy-based strategic processes is delayed in normal aging
Source: PLoS One. 2019 Mar 25;14(3):e0214322. doi: 10.1371/journal.pone.0214322 (PMC6433268; doi:10.1371/journal.pone.0214322)

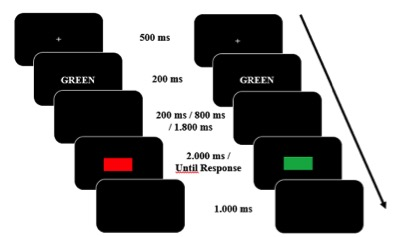

Supplement: S1 Fig — Examples of incongruent (left) and congruent (right) trials used in Experiments 1 and 2. (TIF) [file pone.0214322.s001.tif]

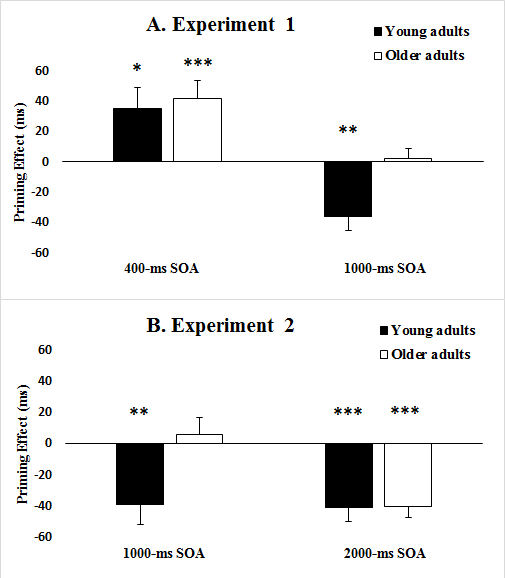

Supplement: S2 Fig — (TIF) [file pone.0214322.s002.tif]

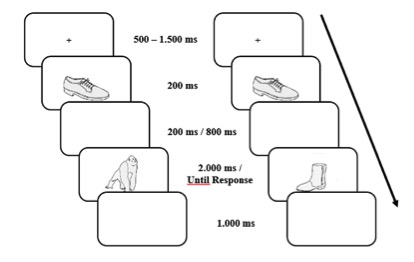

Supplement: S3 Fig — Sequence of events of an incongruent (left) and congruent (right) trial in the Congruency-priming task used in Experiments 3 and 4. (TIF) [file pone.0214322.s003.tif]

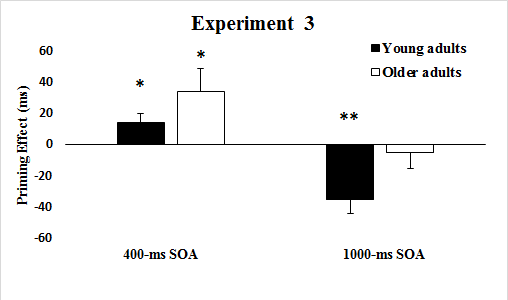

Supplement: S4 Fig — (TIF) [file pone.0214322.s004.tif]

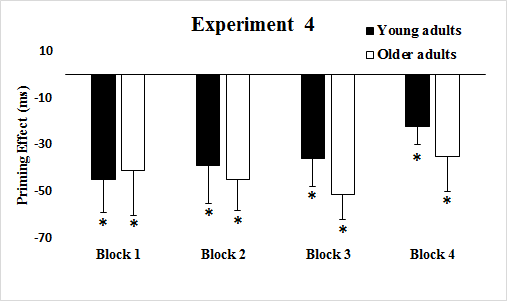

Supplement: S5 Fig — (TIF) [file pone.0214322.s005.tif]
