# Supplementary material for: The implementation of expectancy-based strategic processes is delayed in normal aging
Source: PLoS One. 2019 Mar 25;14(3):e0214322. doi: 10.1371/journal.pone.0214322 (PMC6433268; doi:10.1371/journal.pone.0214322)
Supplement: S1 Appendix — Mean (SD) functional and visual similarity rates in a rating similarity pilot study (1 = not at all similar; 7 = highly similar), for animal and inanimate-object pictures (and their English translations), presented as semantically related prime-target pairs. (PDF) [file pone.0214322.s006.pdf]

S1 Appendix. List of prime-target pairs used in Experiments 3 and 4. Mean (SD) functional and visual similarity rates in a rating similarity pilot study (1= not at all similar; 7 = highly similar), for animal and inanimate-object pictures (and their English translations), presented as semantically related prime-target pairs.

Prime	Target	Functional Similarity	Visual Similarity
Gato (cat)	Perro (dog)	5.75 (1.16)	5.44 (1.12)
Gorila (gorilla)	Mono (monkey)	6.21 (0.94)	5.10 (1.39)
Oveja (sheep)	Cabra (goat)	5.39 (1.07)	5.14 (1.19)
Tigre (tiger)	León (lion)	5.89 (1.03)	5.12 (1.24)
Tornillo (bolt)	Tuerca (screw)	6.38 (0.96)	5.66 (0.99)
Cuchara (spoon)	Tenedor (fork)	6.06 (1.14)	5.23 (1.34)
Taza (cup)	Vaso (glass)	6.58 (0.71)	5.02 (1.61)
Bota (boot)	Zapato (shoe)	6.77 (0.48)	5.01(1.53)
